# Resilience and Service Needs Among African American Caregivers of People With Dementia: A Pilot Mixed-Methods Study

**DOI:** 10.1093/geroni/igaa057.1136

**Published:** 2020-12-16

**Authors:** Heehyul Moon, Sunshine Rote, Hallie Decker, Burton Kelsey, Chelsea Burton, Sharon Moore, Tewsdaay Babicka

**Affiliations:** 1 University of Louisville, Louisville, Kentucky, United States; 2 u of l, Louisville, United States; 3 U OF L, LOUISVILLE, United States; 4 u of l, louisville, United States

## Abstract

African American caregivers face unique challenges and strengths in addressing the needs of dementia care recipients. The purpose of the current study was to explore the roles of the familism and faith and service preferences and needs for African American dementia caregivers. Through collaborative partnerships among the research team, Alzheimer’s’ Association, Area Agency on Aging, and local churches, we obtained focus group and survey data (N=30) from African American dementia caregivers. Most caregivers were female (90%) and older than 51 years and had provided care more than 3 years. CGs showed strong cultural reasons (e.g., faith, duty, paying back) for providing care (range 0-40;M= 34.5; SD= 5.2). Focus groups data, which was audio taped, transcribed, and analyzed by three researchers using content analysis, revealed three major themes related to dementia care experiences and needs. First, caregivers explained positive aspects of caregiving for both the individual caregiver and family (growth in faith, sense of self -efficacy, feeling of gratefulness). Second, caregivers also described negative aspects that pose substantial challenges such as lack of services, lack of balance in life, family conflicts over care, and mistrust based on their previous experiences with existing health care systems. Third, caregivers described their knowledge of dementia and shared their self-care and coping strategies and a need for support group services. The findings show an urgent need to implement culturally responsive services, especially in undeserved areas and populations, for caregivers to maintain or improve their emotional well-being and quality of care as well as family relationships.

